# Efficient Therapeutic Function and Mechanisms of Human Polyclonal CD8^+^CD103^+^Foxp3^+^ Regulatory T Cells on Collagen-Induced Arthritis in Mice

**DOI:** 10.1155/2019/8575407

**Published:** 2019-02-19

**Authors:** Juan Sun, Yiming Yang, Xiaona Huo, Beibei Zhu, Zhenhua Li, Xueyu Jiang, Rufeng Xie, Li Gao, Ying Sun, Huahua Fan, Yongming Zhu, Jie Yang

**Affiliations:** ^1^Institute of Biomedical Sciences, School of Life Sciences, East China Normal University, Shanghai 200241, China; ^2^Blood Engineering Laboratory, Shanghai Blood Center, Shanghai 200051, China

## Abstract

**Objective:**

To investigate the potential therapeutic effect in a rheumatoid arthritis model of stable human CD8^+^ regulatory T cells (hCD8^+^Tregs) induced by TGF-*β*1 and rapamycin (RAPA) *in vitro*.

**Methods:**

Human CD8^+^T cells were isolated from human peripheral blood mononuclear cells and induced/expanded with TGF-*β*1 and RAPA along with anti-CD3/28 beads and IL-2 *in vitro* and harvested as hCD8^+^Tregs. The phenotypes, suppressive characteristics, and stability of the hCD8^+^Tregs in an inflammatory microenvironment were examined *in vitro*. Human CD8^+^Tregs were transfused into an acollagen-induced arthritis (CIA) mouse model, and their therapeutic effects and related mechanisms were investigated.

**Results:**

Human CD8^+^Tregs induced by TGF-*β*1/RAPA showed high expression of Foxp3 and CD103, exhibited vigorous suppression ability, and were stable in inflammatory microenvironments. In CIA mice, the clinical scores, levels of anti-collagen IgG antibody, and cartilage destruction were significantly reduced after adoptive transfusion with hCD8^+^Tregs. Moreover, hCD8^+^Treg treatment significantly reduced the number of Th17 cells, increased the number of CD4^+^IFN-*γ* ^+^T cells, and produced self CD4^+^Foxp3^+^Tregs *in vivo*. In an *in vitro* cell coculture assay, hCD8^+^Tregs significantly inhibited mouse CD4^+^ effector T cell proliferation, induced mouse CD4^+^Foxp3^+^Treg and CD4^+^IFN-*γ* ^+^Th1 cell production, reduced Th17 cell development, and downregulated CD80/86 expression on mature DCs (mDCs).

**Conclusion:**

TGF-*β*1/RAPA can induce hCD8^+^Tregs with stable suppressive characteristics, which could significantly alleviate the severity of CIA based on their stable suppressive ability in an inflammatory microenvironment and further influence the function of other downstream cell subtypes. Human CD8^+^Tregs might be a therapeutic strategy for rheumatoid arthritis.

## 1. Introduction

Rheumatoid arthritis (RA) is a common autoimmune disorder disease characterized by persistent synovitis, systemic inflammation, and autoantibodies; RA develops slowly but causes a poor quality of life for patients [1]. As a well-established model of human RA, collagen-induced arthritis (CIA) in mice shares many key features with RA in humans, such as synovitis, erosion of both bone and cartilage, and MHC II molecular-linked susceptibility [2, 3], and is commonly used to study the cause of RA and/or develop new therapies. Previous studies have shown that regulatory T cells (Tregs) in autoimmune disease (AID) patients are deficient or dysfunctional [4] and that transfusing Tregs has an efficient therapeutic function in AID models [5, 6]. Therefore, Tregs have been considered promising candidates as cellular therapy for RA.

Tregs are potent suppressors of T cell responses *in vitro* and *in vivo* [7] and play an essential role in immune regulation and autoimmune disease prevention. However, the number of natural CD4^+^Foxp3^+^Tregs (nTregs) is very low, which severely restricts their clinical application. Moreover, the suppression function of natural CD4^+^Tregs is very unstable, especially in inflammatory conditions, in which the cells show attenuation of Foxp3 expression, conversion into Th17 cells, and failure to affect established diseases [8, 9]. Therefore, it is desirable to find an approach that can expand stable Tregs for cellular therapy.

Fortunately, recent reports have indicated that there are CD4^+^Treg subtypes and CD8^+^Treg subtypes in the “Treg family” [10]. As a new Treg subtype, CD8^+^Tregs also express a high level of Foxp3, a commonly known key marker of Tregs, and play an important role in the maintenance of self-tolerance, independent of CD4^+^T cells [11], inducing the conversion of CD4^+^Foxp3^+^Tregs to Th17 [12]. Thus, CD8^+^Tregs appear to be a better therapeutic cell candidate for AID treatment.

Several approaches for the induction of antigen-specific CD8^+^Tregs *in vitro* have been reported [13]. However, no reliable protocol for the ex vivo induction of human polyclonal CD8^+^Foxp3^+^Tregs is currently available. TGF-*β*1 was reported to induce Foxp3^+^Tregs from CD4^+^CD25^−^T cells [14], and TGF-*β*1 plus low-dose rapamycin (RAPA) synergize to upregulate Foxp3 expression and suppress human T cell responses [15]. Therefore, in this study, TGF-*β*1 plus RAPA was used to induce and adequately expand hCD8^+^Foxp3^+^Tregs from hCD8^+^T cells *in vitro*, and the characteristics and stability of these induced hCD8^+^Foxp3^+^Tregs were identified. Furthermore, the therapeutic function of hCD8^+^Foxp3^+^Tregs in CIA mice after adoptive transfusion was confirmed, and the related mechanism of hCD8^+^Foxp3^+^Treg treatment was revealed.

## 2. Materials and Methods

### 2.1. The Induction and Expansion of CD8^+^Tregs

This study was approved by the Ethics Committee of Shanghai Blood Center (Permit Number: SBC-IRB-2013-07). All human donors were enrolled in the study after providing written informed consent. Human CD8^+^T cells were derived from peripheral blood mononuclear cells (PBMCs) collected from healthy blood donors (Shanghai Blood Center, Shanghai, China) and sorted using a human CD8^+^T cell isolation kit (Miltenyi Biotec, Bergisch Gladbach, Germany). Briefly, 1 × 10^5^/mL hCD8^+^T cells were induced with TGF-*β*1 (50 ng/mL, PeproTech) and RAPA (100 nM, LC Laboratories) plus anti-CD3/CD28-coated expansion beads (cells : beads = 1 : 4, Invitrogen) and IL-2 (1600 IU/mL, PeproTech) in 1% human AB serum (from healthy blood donors, Shanghai Blood Center, China) in RPMI 1640. Cells were cultured in 96-well round plates and harvested after 9 days.

### 2.2. Flow Cytometry Analysis

Human CD8^+^Tregs were stained with anti-human CD28, CD103, and PD-1-PE (BD Pharmingen). For intracellular staining, cells were fixed, permeabilized, and stained with anti-human Foxp3, IL-2, IL-10, IL-17, and IFN-*γ*-PE (BD Pharmingen) after stimulation by phorbol-myristate-acetate (PMA, 25 ng/mL, Sigma), ionomycin (ION, 1 *μ*g/mL, Sigma), and monensin (MON, 2 *μ*g/mL, Sigma) for 4 hours. The expression of surface or intracellular markers was measured by FACS.

### 2.3. Measurement of Cytokines by Cytometric Bead Array (CBA)

Human CD8^+^Tregs (1 × 10^6^ cells/mL) were washed twice and stimulated with PMA (25 ng/mL) and ION (1 *μ*g/mL) in 1% human AB serum RPMI 1640 for 24 hours. The same culture medium as that used for the control was used. Following activation, supernatants were harvested for the CBA assay (BD Pharmingen) to examine the secretion of TGF-*β*1, IL-17A, and IFN-*γ* according to the manufacturer's protocol.

### 2.4. *In Vitro* Treg Stability in Inflammation

Human CD8^+^Tregs (5 × 10^5^ cells/mL) were activated with anti-CD3/CD28 expansion beads (cells : beads = 1 : 1) with the following inflammatory mixtures: inflammatory mixture-A (Infla-A) contained IL-2 (10 IU/mL), IL-1*β* (10 ng/mL), IL-6 (4 ng/mL), and TGF-*β*1 (5 ng/mL), and inflammatory mixture-B (Infla-B) contained IL-2 (10 IU/mL), IL-21 (25 ng/mL), IL-23 (25 ng/mL), and TGF-*β*1 (5 ng/mL). All inflammatory factors were purchased from PeproTech. Human CD8^+^Tregs were cultured with IL-2 (10 IU/mL) as the control. Supernatants were harvested and evaluated by CBA after 6 days. Meanwhile, the expression levels of Foxp3, IL-2, IL-17A, and IFN-*γ* were determined on days 3, 6, and 9 by FACS after hCD8^+^Tregs were cultured with Infla-A or -B.

### 2.5. The Stability of hCD8^+^Tregs *In Vitro* after Removing Induction Factors or Decreasing Expansion Factors

Human CD8^+^Tregs were washed twice to remove the induction factors (TGF-*β*1 and RAPA), cultured in normal expansion conditions (IL-2, 1600 IU/mL; anti-CD3/CD28 expansion beads, cells : beads = 1 : 4), 1/2 normal expansion conditions (IL-2, 800 IU/mL; cells : beads = 1 : 2), or 1/10 normal expansion conditions (IL-2, 160 IU/mL; cells : beads = 1 : 0.4). Cells cultured with a conventional dose of TGF-*β*1/RAPA in expansion conditions were considered controls. Foxp3 expression was determined on days 3, 6, and 9 by FACS to reflect Treg stability.

### 2.6. Collagen-Induced Arthritis (CIA) Model

The animal study was approved by the Institutional Animal Care and Use Committee of Shanghai Blood Center (Permit Number: SBC-IRB-2016-02). All animal studies involving mice were conducted in strict accordance with the recommendations in the guidelines of the Institutional Animal Care and Use Committee of the Chinese Association for Laboratory Animal Sciences. Mice were maintained in specific pathogen-free conditions and received standard laboratory food and water. All surgeries were performed under diethyl ether anesthesia, and the mice were sacrificed using carbon dioxide, which minimizes animal suffering. Six to eight-week-old male DBA1/J mice were purchased from SLACAS (Shanghai, China). CIA mice were immunized twice on days 0 and 21 with bovine type II collagen (CII, Chondrex) [16]. After 28 days, the onset of CIA was confirmed and examined every two days using the scoring system, as previously described [17]. An arthritis score (range, 0-16) was assigned to each mouse by summing the scores of each paw.

### 2.7. Adoptive Transfusion of hCD8^+^Tregs

On day 28 after the first CII immunization, CIA mice were stochastically divided into 3 groups, which were transfused intravenously with 4 × 10^6^ hCD8^+^Tregs, 4 × 10^6^ PBMCs, and PBS only and termed the hCD8^+^Treg group, the PBMC group, and the control group, respectively. Mice were examined every two days for the next 4 weeks.

### 2.8. CD8^+^Treg Survival in CIA Mice

On day 28 after the first CII immunization, 4 × 10^6^ hCD8^+^Tregs labeled with CFSE were transfused intravenously into CIA mice. After 3 days of transfusion, the mice were sacrificed, and the feet, spleen, lymph node, and blood cells were collected, labeled with PE anti-human CD8 and APC anti-human Foxp3 antibodies, and then evaluated by FACS. CFSE^+^CD8^+^ cells were considered survival hCD8^+^Tregs in CIA mice, and the percentage of Foxp3 expression was also examined to indicate hCD8^+^Treg stability after transfusion *in vivo*.

### 2.9. Histology Evaluation of CIA

Mice were sacrificed on day 56 after the first CII immunization; the paws were collected, and the joint tissues were fixed in 4% paraformaldehyde, decalcified in the EDTA, and embedded in paraffin. The sections were then dewaxed using xylene and dehydrated through an alcohol gradient. Endogenous peroxidase activity was quenched with methanol/3% H_2_O_2_. Sections were stained in a routine manner with H&E and Safranin O-Fast Green.

### 2.10. Anti-bovine CII IgG Detection in Serum

Mice were sacrificed individually on day 56 after the first CII immunization, and the serum was maintained at −70°C until use. The content of the anti-bovine CII IgG antibody was tested by ELISA (Chondrex) according to the manufacturer's instructions.

### 2.11. Detection of Cell Subsets in Mouse Spleen and mRNA Expression in Mouse Paws

Mice were sacrificed 72 hours after hCD8^+^Treg transfusion, and splenocytes were isolated and stimulated with PMA (25 ng/mL), ION (1 *μ*g/mL), and MON (2 *μ*g/mL) in RPMI 1640 supplemented with 10% fetal bovine serum (Invitrogen) for 4 hours. Cells were labeled with FITC anti-mouse CD4 (mCD4), and intracellular cytokines were stained with PE anti-mouse Foxp3, IL-17A, and IFN-*γ*. The percentages of Foxp3^+^, IL-17A^+^, and IFN-*γ* ^+^ cells in mCD4^+^T cells were detected by FACS.

For mRNA detection, the mice were sacrificed 72 hours after transfusion, and the paws were collected. The tissues were ground in liquid nitrogen, and mRNA was subsequently extracted, transcribed to cDNA, and evaluated by q-PCR.

### 2.12. Suppression Assay

Mouse CD4^+^CD25^−^T (mCD4^+^CD25^−^) cells were isolated from splenocytes, labeled with CFSE, and stimulated with anti-mouse CD3/CD28 expansion beads (cells : beads = 1 : 1), mouse IL-2 (40 IU/mL), and bovine CII (100 ng/mL). Human CD8^+^Tregs were then added at different doses (hCD8^+^Tregs : mCD4^+^CD25^−^ effector T cells = 1 : 1, 1 : 2, 1 : 4, and 1 : 16). After 3 days of coculture, cells were detected by FACS, and the CFSE dilution was analyzed to calculate the inhibition ability. CD8^+^Treg inhibition ability was evaluated using human allogeneic and autogeneic assays. Human CD4^+^CD25^−^T cells were purified from the same donor with CD8^+^Treg (autogeneic) or not (allogeneic), labeled with CFSE, stimulated with anti-human CD3/CD28 expansion beads (cells : beads = 1 : 1) and human IL-2 (40 IU/mL), cocultured at different ratios (hCD8^+^Tregs : hCD4^+^CD25^−^ effector T cells = 1 : 1, 1 : 4, 1 : 16, or 1 : 64), and evaluated by FACS after 3 days.

### 2.13. Influence on mCD4^+^CD25^−^ Cell Differentiation and Apoptosis

Purified mCD4^+^CD25^−^T cells were cocultured with hCD8^+^Tregs, as described above, at a ratio of 1 : 1 for 4 days. Mouse CD4^+^CD25^−^T cells were selected by staining with FITC-anti-mouse CD4. Foxp3^+^, IFN-*γ* ^+^, and IL-17A^+^ cells in mCD4^+^CD25^−^T cells were evaluated to determine mCD4^+^CD25^−^T cell differentiation. The mCD4^+^CD25^−^T cells were stained with Annexin V and propidium iodide (PI) to measure apoptosis.

### 2.14. Influence of CD80/CD86 Expression on DCs

Mouse DCs (mDCs) derived from bone marrow were cultured in medium containing GM-CSF (20 ng/mL, PeproTech) and IL-4 (1 ng/mL, PeproTech). On day 6, DCs were loaded with CII (10 *μ*g/mL, Chondrex), matured with LPS (100 ng/mL, Sigma) for 16 hours, and subsequently cocultured with or without 5 × 10^4^ hCD8^+^Tregs (at the ratio of Tregs : mDCs = 5 : 1) for 24 hours. In the same manner, mCD4^+^CD25^−^T cells cocultured with mDCs were used as controls. APC anti-mouse CD11c was used for staining to select DCs, and CD80/CD86 expression in mDCs was analyzed.

### 2.15. Statistical Analysis

The data are presented as the mean ± standard error of the mean (SEM). All statistical analyses were performed using GraphPad software version 6.0 for Windows. The intergroup analysis was performed using Student's *t*-test. *P* values below 0.05 were considered significant.

## 3. Results

### 3.1. Human CD8^+^CD103^+^Foxp3^+^Treg Cells Can Be Induced by TGF-*β*1/RAPA with Suppressive Characteristics *In Vitro*

The clinical application of Tregs requires a large amount of highly suppressive and stable Tregs. The characteristics of hCD8^+^Tregs are still not clear, although some studies have noted this new type of Tregs. Therefore, in this study, the phenotypes and suppression ability of ex vivo polyclonal induced/expanded hCD8^+^Tregs were analyzed.

**Figure 1:**
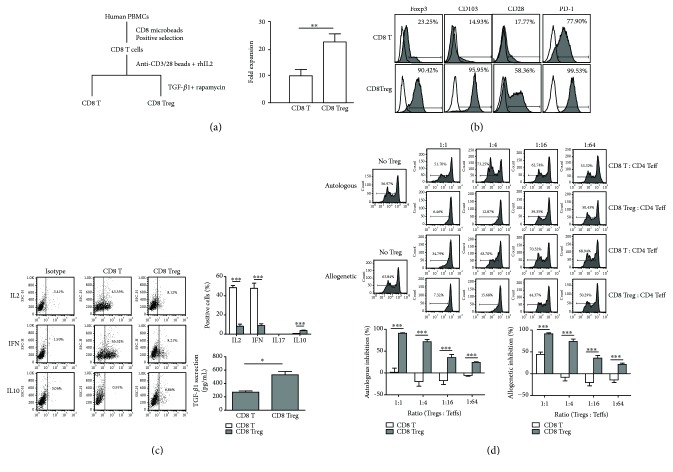
Phenotypes, cytokines, and suppressive ability of induced/expanded human CD8^+^Tregs *in vitro*. (a) The induction and expansion of human CD8^+^Tregs. A total of 1 × 10^5^ CD8^+^T cells derived from human PBMCs were sorted and induced with TGF-*β*1 and RAPA plus anti-CD3/CD28 expansion beads and IL-2 for 9 days. hCD8^+^ T cells were derived from the same CD8^+^ T cells using expansion beads and IL-2, but without TGF-*β*1 or RAPA. Expended CD8^+^ T and CD8^+^Treg cells were collected and counted with a hemocytometer under a microscope, and cell numbers and expansion folds were calculated. (b) Phenotypes of induced human CD8^+^Tregs analyzed by FACS. Human CD8^+^Tregs and T cells were collected and stained with anti-human CD28, CD103, and PD-1-PE. Foxp3 was also measured after the cells were fixed, permeabilized, and stained with anti-human Foxp3-PE. (c) Cytokine expression in induced human CD8^+^Tregs. On day 9, human CD8^+^Tregs were harvested, washed twice with PBS, and stimulated with PMA, ION, and MON for 4 hours; then, intracellular cytokines, such as IL-2, IL-10, IL-17, and IFN-*γ*, were evaluated by FACS. Meanwhile, human CD8^+^Tregs and T cells were treated with the above method, but without MON stimulation, for 24 hours, and TGF-*β*1 secretion in the supernatant was evaluated by ELISA. (d) Inhibition of human CD8^+^Tregs on autologous and allogeneic human CD4^+^CD25^−^ effector T cells. Briefly, 5 × 10^4^ human CD4^+^CD25^−^ effector T cells (Teffs) labeled with CFSE were stimulated with anti-human CD3/CD28 expansion beads and IL-2 and cocultured with human CD8^+^ T cells or human CD8^+^Tregs for 3 days (CD8^+^Treg : CD4^+^Teff = 1 : 1, 1 : 4, 1 : 16, and 1 : 64). For autogenic inhibition, purified CD4^+^CD25^−^ effector T cells and expanded CD8^+^T and CD8^+^Tregs were derived from the same donor, while CD4^+^CD25^−^ effector T cells were purified from different donors with CD8^+^ T and CD8^+^Treg cells in allogeneic inhibition assay. CFSE dilutions were investigated to reflect CD4^+^Teff proliferation. Inhibition ability was calculated by (control Teff proliferation − Teff with Treg proliferation)/(control Teff proliferation) × 100%. Each mean represents at least 3 individual samples; the bars indicate the mean ± SEM; ^∗^ *P* < 0.05, ^∗∗^ *P* < 0.01, and ^∗∗∗^ *P* < 0.001.

As shown in Figure 1(a), hCD8^+^Tregs were induced by TGF-*β*1 and RAPA from hCD8^+^T cells and expanded *in vitro*; the cells expressed high levels of Foxp3, CD25, and CD103 (Figure 1(b)). Meanwhile, these hCD8^+^Tregs had stable activity, and the apoptosis percentage was only 6.40 ± 1.05% (not shown). Compared with expanded hCD8^+^T cells as controls, the induced hCD8^+^Tregs expressed lower levels of IFN-*γ* and IL-2 and increased IL-10. In particular, the secretion of TGF-*β*1 was significantly upregulated (Figure 1(c)). Moreover, the hCD8^+^Tregs were IL-17A-negative, and there was no IL-17A secretion in the culture supernatant.

To investigate the stable and potent regulatory function of induced hCD8^+^Tregs, the cells were cocultured with CFSE-labeled autogenetic or allogeneic human CD4^+^CD25^−^ (hCD4^+^CD25^−^) effector T cells at different ratios *in vitro*. The data showed that hCD8^+^ Tregs had potent suppressive activity on both autogenetic and allogeneic effects of T cell proliferation, and there was no significant difference between autogenetic and allogeneic T cells (Figure 1(d)). Therefore, Treg suppressive functions were maintained, whether autogenetic or not, and this finding is the basis for the wide application of Tregs in cell therapy.

### 3.2. Human CD8^+^CD103^+^Foxp3^+^Tregs Show Stable Suppressive Characteristics without Induction Factors or in an Inflammatory Microenvironment *In Vitro*

**Figure 2:**
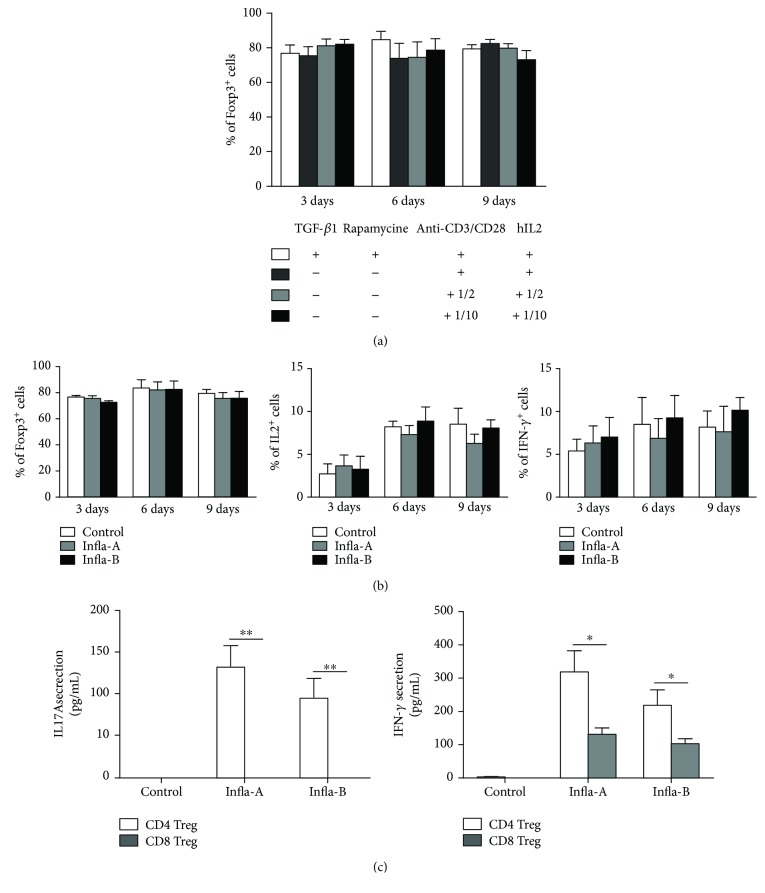
Stability of human CD8^+^Tregs without induction factors or in an inflammatory microenvironment *in vitro*. (a) Foxp3 expression when induction factors were removed *in vitro*. Compared with normal induction/expansion conditions, the level of Foxp3 expression in induced human CD8^+^Tregs when induction factors (TGF-*β*1 and RAPA) were removed or induction factors were removed with only 1/2 or 1/10 of normal expansion factors. (b) Stability of induced human CD8^+^Treg cells in two different inflammatory conditions *in vitro*. Two inflammatory microenvironments were modeled with different inflammatory cytokine mixtures (Infla-A: IL-2 10 IU/mL, IL-1*β* 10 ng/mL, IL-6 4 ng/mL, and TGF-*β*1 5 ng/mL; Infla-B: IL-2 10 IU/mL, IL-21 25 ng/mL, IL-23 25 ng/mL, and TGF-*β*1 5 ng/mL). Then, CD8^+^Tregs were cultured in these two different inflammatory microenvironments, and IL-2, Foxp3, and IFN-*γ* expression of human CD8^+^Tregs was evaluated on days 3, 6, and 9 by FACS. (c) Compared with human natural CD4^+^Tregs, IFN-*γ* and IL-17A secretion in the supernatant on day 6 was investigated by CBA. Human natural CD4^+^CD25^+^Treg cells were purified from PBMCs and expanded with anti-CD3CD8/CD28 beads plus RAPA *in vitro*. After 9 days of expansion, CD4 Treg cells were harvested and washed. Expended CD8^+^Tregs and CD4^+^Tregs were cultured in the above two inflammation conditions (Infla-A and Infla-B) for 6 days. The supernatant was collected, and IL-17A and IFN-*γ* secretion was evaluated with CBA. Each mean represents at least 3 individual samples; the bars indicate the mean ± SEM; ^∗^ *P* < 0.05, ^∗∗^ *P* < 0.01, and ^∗∗∗^ *P* < 0.001.

Many studies have shown that natural CD4^+^ regulatory T cells (nTregs) are unstable and convert into Th17 cells when they encounter inflammation, which is harmful for use in clinical therapy [8, 9]. Thus, whether induced hCD8^+^Foxp3^+^Tregs can maintain their suppressive characteristics in the microenvironment without induction factors or within inflammatory factors *in vitro* was investigated. Our research found that after hCD8^+^Tregs were induced by TGF-*β*1/RAPA for 9 days, the Foxp3 expression of these hCD8^+^Tregs was still maintained without induction factors after subsequent culture and even exhibited a reduced need for stimulation beads and IL-2 (Figures 2(a) and S1). More importantly, the results showed that compared with the control Tregs under normal conditions, the levels of Foxp3, IL-2, and IFN-*γ* expression on hCD8^+^Foxp3^+^Tregs in two different inflammatory conditions (imitated with different inflammatory cytokine mixtures) on days 3, 6, and 9 were not significantly different (Figures 2(b) and S2). Additionally, hCD8^+^Foxp3^+^Tregs did not completely express IL-17A in the above two inflammation conditions. Meanwhile, compared with hCD4^+^Tregs, IL-17A and IFN-*γ* secretion in the culture supernatant of hCD8^+^Tregs stimulated by different inflammatory cytokines on day 6 was lower (Figure 2(c)).

### 3.3. After Adoptive Transfusion, Human CD8^+^Tregs Can Survive and Are Stable in CIA Mice

**Figure 3:**
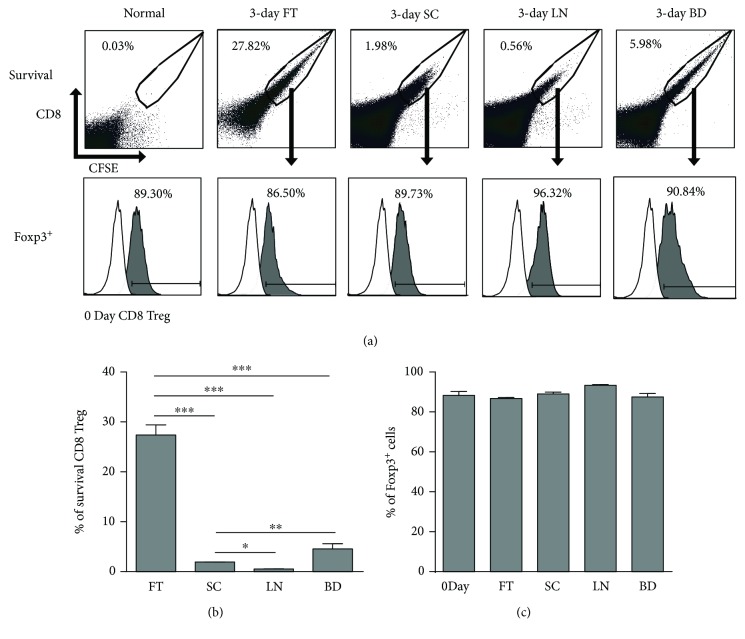
Survival and stability of induced human CD8^+^Tregs transfused into CIA mice. Briefly, 4 × 10^6^ human CD8^+^Tregs labeled with CFSE were transfused into established CIA mice on day 28 by *i.v.* After 72 hours of adoptive infusion, the mice were sacrificed, and the cells in their blood (BD), spleen cells (SC), lymph nodes (LN), and paws (foot, FT, minced, and digested) were harvested and labeled using PE-conjugated anti-human CD8. The cells were subsequently fixed, permeabilized, and labeled with anti-human APC-Foxp3 and evaluated by FACS. CD8^+^CFSE^+^ cells were considered surviving CD8^+^Tregs, and the percentage of Foxp3-positive cells in CFSE^+^CD8^+^ surviving cells was investigated to evaluate the stability of CD8^+^ Tregs in CIA. (a) Survival of human CD8^+^Tregs after transfusion into CIA mice for 72 hours. Representative flow data showed surviving human CD8^+^CFSE^+^Treg (above) and Foxp3 expression in surviving CD8^+^Tregs (below). (b) Quantitative analysis of surviving human CD8^+^Tregs *in vivo* after transfusion for 72 hours. (c) Quantitative analysis of Foxp3 expression in surviving human CD8^+^Tregs *in vivo* after transfusion for 72 hours. Each mean represents data from 5 mice in each group; the bars indicate the mean ± SEM; ^∗^ *P* < 0.05, ^∗∗^ *P* < 0.01, and ^∗∗∗^ *P* < 0.001.

As described above, human CD8^+^Tregs were stable when they encountered different inflammatory factors *in vitro*. However, it was still necessary to investigate the stability of human CD8^+^Tregs and their survival under complex inflammatory conditions, similar to CIA onset in mice. Thus, the survival of hCD8^+^Tregs and their Foxp3 expression in different tissues were tested in established CIA mice after hCD8^+^Tregs were transfused into mice for 72 hours. The FACS results indicated that hCD8^+^Tregs were present in the feet (27.40 ± 2.03%), spleen (1.90 ± 0.05%), lymph node (0.50 ± 0.04%), and blood (4.55 ± 1.03%) of CIA mice, as shown in Figures 3(a) and 3(b). The percentage of Foxp3^+^ cells in the surviving hCD8^+^Tregs remained for at least 72 hours *in vivo*, revealing that hCD8^+^Tregs are stable after transfusion in CIA mice.

### 3.4. Human CD8^+^Tregs Induced by TGF-*β*1/RAPA Display Effective Anti-arthritic Activities in CIA Mice

**Figure 4:**
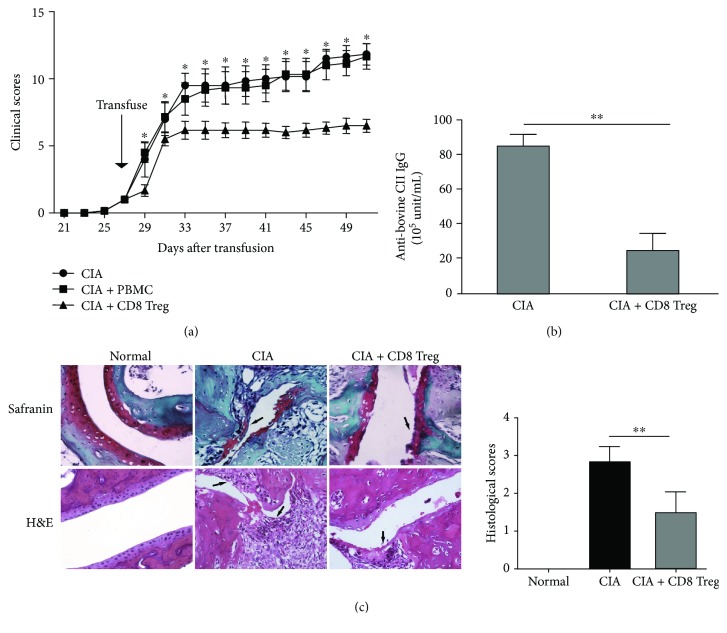
Therapeutic functions of human CD8^−^Tregs on collagen-induced arthritis in mice. Briefly, human 4 × 10^6^ CD8^+^Tregs were adoptively transferred into established CIA mice on day 28 after the first immunization. Mice were scored for clinical signs of arthritis in the limb joints by macroscopic examination three times a week. Limb joint arthritis was assessed by an established scoring system. A series of arthritic indices were examined. (a) Clinical scores. Arthritic scores analyzed from day 21 in each group (untreated CIA mice, PBMC-treated mice, and CD8^+^Treg-treated mice) during the observation period are shown. Each group had at least 6 individual mice; ^∗^ compared with CIA mice and the PBMC group by unpaired *t*-tests. (b) Anti-bovine CII IgG detection in serum. Sera were collected on day 56 to measure the content of anti-CII-specific IgG antibody by ELISA. Each mean represents at least 3 individual samples; the bars indicate the mean ± SEM; ^∗^ *P* < 0.05 and ^∗∗^ *P* < 0.01. (c) Histology evaluation. Hind paws were collected on day 56. Images of the affected paws were captured, and tissues were stained with Safranin O-fast green (upper, showing cartilage erosion) and H&E (lower, showing synovial joint inflammation) (magnification, 200x). The arrows indicate representative areas of cartilage erosion (upper) and synovial joint inflammation (lower); statistics results were calculated from 3 individual mice.

The CIA model of arthritis is a well-established method for evaluating therapeutic interventions in autoimmune arthritis. Briefly, 4 × 10^6^ hCD8^+^Tregs or PBMC were transferred into CIA mice on day 28 after the first CII immunization, and then the arthritic index and histopathology were examined. During the 4-week observation period, compared with mice that were injected with commensurable PBMCs or the untreated control CIA mice, mice infused with 4 × 10^6^ hCD8^+^Tregs had lower arthritis scores. On day 51, the scores of hCD8^+^Treg-treated mice were stably controlled at 6.50 ± 0.48, while those of both the PBMC-treated and untreated groups increased remarkably to approximately 11.50, with no significant difference between them. Thus, the statistical analysis showed that hCD8^+^Tregs had significant suppressive ability in CIA mice (Figure 4(a)). Meanwhile, the levels of CII-specific IgG antibodies in the serum from different treatment groups on day 56 were also investigated, and hCD8^+^Treg treatment significantly reduced CII-specific IgG antibody secretion, as shown in Figure 4(b).

Because lymphocyte T cell infiltration and cartilage erosion reflect the damage caused by disease, histological differences in normal DBA1/J, control CIA mice, and hCD8^+^Treg-treated CIA mice were assessed on day 56 after the first CII immunization (Figure 4(c)). Examination of pathological specimens revealed that compared with those of control CIA mice, the joints of mice treated with hCD8^+^Tregs showed a remarkable decrease in the destruction of cartilage and infiltration of inflammatory cells.

### 3.5. Human CD8^+^Tregs Can Influence Splenic CD4^+^T Subtypes and the Expression of Various Cytokine mRNAs in the Feet of CIA Mice

**Figure 5:**
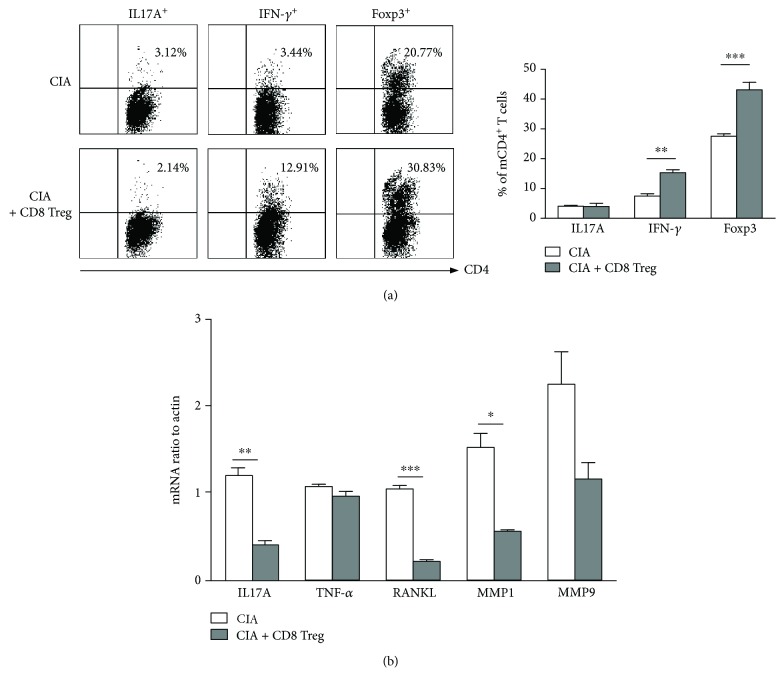
The mechanisms of human CD8^+^Treg-controlled CIA mice *in vivo*. (a) The influence of human CD8^+^ Tregs on splenic CD4^+^ T cell subtypes in CIA mice. Splenic cells were isolated, and the CD4^+^ T cell subtypes were determined after 72 hours of transfer. The levels of Foxp3, IL-17A, and IFN-*γ* in mouse CD4^+^T cells were evaluated by FACS. The right graph shows the representative FACS data, and the left graph indicates the statistical results. (b) mRNA expression of cytokines and related factors in CIA mouse feet. CIA mice were sacrificed 72 hours after human CD8^+^Treg transfusion; then, their paws were minced and digested, and the cells were collected. Subsequently, mRNA was measured by real-time PCR. The results are expressed as the mean ± SEM of at least three independent experiments; ^∗^ *P* < 0.05, ^∗∗^ *P* < 0.01, and ^∗∗∗^ *P* < 0.001, compared with the control group by unpaired *t*-tests.

Mouse CD8^+^Treg transfusion can induce conventional CD4^+^Foxp3^+^T cells and concurrently suppress effector T cell expansion to alleviate disease severity in EAE and skin allograft mouse models [13, 18]. Therefore, further investigation of the potential therapeutic functional mechanisms of hCD8^+^Tregs on CIA mice was conducted. In established CIA mice, the CD4^+^T cell subtype in mouse spleens after hCD8^+^Treg treatment for 3 days was evaluated by FACS. Human CD8^+^Treg therapy significantly induced the generation of mouse self-CD4^+^Foxp3^+^Tregs and CD4^+^IFN-*γ* ^+^T cells in the spleen (Figure 5(a)). Additionally, after transfusion for 3 days, the mRNA expression of IL-17, TNF-*α*, receptor activator for nuclear factor-*κ*B ligand (RANKL), matrix metalloproteinase-1 (MMP1), and MMP9 in the feet of hCD8^+^Treg-treated and untreated CIA mice was determined by real-time PCR. Furthermore, hCD8^+^Treg treatment obviously reduced the mRNA expression of IL-17A, RANKL, and MMP1, while TNF-*α* and MMP9 showed no significant change (Figure 5(b)).

### 3.6. Human CD8^+^Tregs Can Suppress mCD4^+^CD25^−^T Cell Proliferation, Influence mCD4^+^CD25^−^ T Cell Differentiation, and Reduce CD80/86 Expression on mDCs

**Figure 6:**
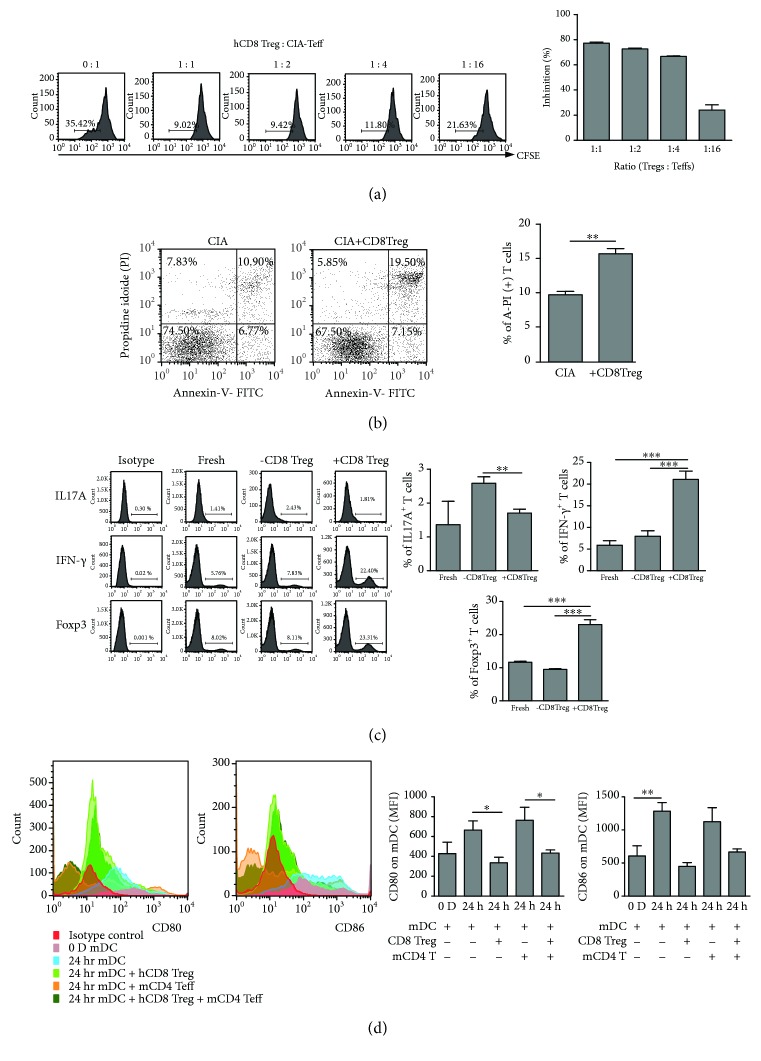
The therapeutic function of human CD8^+^Tregs on CD4^+^CD25^−^ effector T cells and mature DCs of CIA mice in an *in vitro* cell coculture assay. (a) Suppression ability of human CD8^+^Tregs on CD4^+^CD25^−^ effector T cells of CIA mice. Mouse CD4^+^CD25^−^T cells were purified from splenic cells from CIA mice on day 35 after the first immunization. CFSE-labeled mCD4^+^CD25^−^ effector T cells (Teffs) were cocultured with or without hCD8^+^Tregs at different ratios (Treg : Teff = 1 : 1, 1 : 2, 1 : 4, and 1 : 16) under the stimulation of anti-mouse CD3/CD28 expansion beads (mCD4^+^T : bead = 1 : 1) and CII (100 ng/mL) for 3 days. A CFSE dilution was investigated to reflect mCD4 proliferation. hCD8^+^Treg inhibitory ability was calculated as (control Teff proliferation − Teff with Treg proliferation)/(control Teff proliferation) × 100%. (b) Associated apoptosis/death induction of CD8^+^Tregs on CD4^+^CD25^−^ effector T cells of CIA mice. Mouse CD4^+^CD25^−^ effector T cells were cocultured with or without CD8^+^Treg cells for 72 hours. Annexin V-FITC and propidium iodide (PI) were used to label the cells, which were evaluated by FACS. The Annexin V^+^PI^+^ cells were considered to indicate apoptotic/dead cells. Cells from 3 individual mice were tested, and a representative graph is shown on the left. (c) The influence of human CD8^+^Tregs on CD4^+^CD25^−^ T cell differentiation in mice. At the ratio of Treg : Teff of 1 : 1, mouse CD4^+^CD25^−^ T cells were selected, and the levels of Foxp3, IL-17A, and IFN-*γ* were evaluated to investigate the influence of human CD8^+^Tregs on CIA mouse CD4^+^CD25^−^ T cell differentiation. (d) CD80 and CD86 expression in mature mouse DCs. DCs from CIA mouse bone marrow were sorted, matured using LPS (100 ng/mL), and cocultured with or without human CD8^+^Tregs for 24 hours. Then, CD80 and CD86 expression in mDCs was evaluated. The results are expressed as mean ± SEM of at least three independent experiments; ^∗^ *P* < 0.05, ^∗∗^ *P* < 0.01, and ^∗∗∗^ *P* ≤ 0.001, compared with the control group by unpaired *t*-tests.

To further demonstrate the influence of hCD8^+^Tregs on mouse CD4^+^CD25^−^effector T cells from CIA mice (as CIA-Teffs in Figure 6(a)), hCD8^+^Tregs were cocultured with CIA-Teffs for 3 days *in vitro*. CIA-Teffs underwent proliferation after stimulation by anti-mouse CD3/CD28 expansion beads, but this proliferation was suppressed by the presence of hCD8^+^Tregs. These data showed that hCD8^+^Tregs exhibited potent suppressive activity against CIA-Teff proliferation and induced apoptosis (Figures 6(a) and 6(b)).

Moreover, hCD8^+^Tregs also affected the differentiation of mCD4^+^CD25^−^T cells. As shown in Figure 6(c), hCD8^+^Tregs induced the production of mouse self-CD4^+^Foxp3^+^Tregs and CD4^+^IFN-*γ* ^+^Th1 cells and inhibited the development of Th17 cells in an *in vitro* cell coculture assay, revealing that the disease process was controlled through these pathways.

Additionally, as antigen-presenting cells, DCs play an important role in the immune response. In an *in vitro* coculture assay, hCD8^+^Tregs could downregulate CD80 and CD86 expression in mature DCs from CIA mice, whether mCD4^+^CD25^−^ effector T cells were present (Figure 6(d)).

## 4. Discussion

Recently, several studies have revealed that CD4^+^Tregs play a critical role in cell therapy in AID models [2, 3]. However, these studies have also indicated that nTregs have plasticity when they encounter inflammatory factors and lose their suppression function to modify established disease [8, 9]. Fortunately, recent reports have indicated that there are CD4^+^Treg subtypes and CD8^+^Treg subtypes in the Treg family [10]. Unlike CD4^+^Tregs, the characteristics and function of CD8^+^Tregs remain unclear. Several subsets with different markers, such as CD8^+^Fopx3^+^, CD8^+^CD28^−^, or CD8^+^CD103^+^ T cells, are generally considered to be suppressive CD8^+^T cells or CD8^+^Tregs, all of which have been shown to control immune responses in preclinical and clinical models [12, 13]. However, the exact properties of the CD8^+^ suppressor/regulatory T cells, as well as their suppressive function and related mechanism in allogeneic transplantation, remain elusive.

Recently, some studies have provided several approaches for the induction of antigen-specific CD8^+^Tregs with suppressive functions [13, 19], but there is currently no reliable protocol for the ex vivo induction and large-scale expansion of human polyclonal CD8^+^CD103^+^Foxp3^+^Tregs to investigate their suppression ability in immune disorders. Additionally, several studies have shown that TGF-*β*1 and RAPA can induce CD4^+^T cells into CD4^+^Foxp3^+^Tregs. In this study, we successfully established an effective method to induce/expand human CD8^+^Tregs using TGF-*β*1 plus RAPA *in vitro*. These novel induced hCD8^+^Tregs expressed a stable and high level of Foxp3, CD103, and PD-1, showed no secretion of IL-17A even in an inflammatory microenvironment, and survived *in vivo*. In addition, the induced CD8^+^Tregs possessed potent regulatory activity *in vitro* and *in vivo*. Human CD8^+^Tregs reduced disease severity in the CIA model, induced mouse self-CD4^+^Foxp3^+^Tregs and CD4^+^IFN-*γ* ^+^ cells, reduced mouse Th17 cells, and influenced mouse mRNA expression in CIA paws. TGF-*β*1 and RAPA could induce CD8^+^T cells to stably express Treg characteristics.

Suppression ability is the most important functional character of Tregs, which makes it a unique subtype distinct from other T cells. In our research, we found that hCD8^+^Tregs induced by TGF-*β*1/RAPA had potent suppressive activity on both autogenetic and allogeneic effects of T cell proliferation. More startling, these induced hCD8^+^Tregs could also inhibit the proliferation of mouse CD4^+^ effector T cells. Meanwhile, in the cell survival assay, we found that hCD8^+^Treg adoptive transfusion into CIA mice was not cleared immediately and instead remained for some days to play an effective suppressive function during the disease process. These exciting findings suggest that hCD8^+^Tregs would be a stable suppressive functional candidate for cellular therapy, which would provide substantial possibilities for autogenetic or allogeneic clinical therapy.

In some mouse models, it was reported that CD8^+^Treg transfusion could alleviate disease severity and improve mouse self-immune system remodeling. Some studies showed that CD8^+^Treg transfusion induced conventional CD4^+^Foxp3^+^T cells to alleviate disease severity in EAE and skin allograft models [18]; others also indicated that antigen-specific CD8^+^Treg cell could induce CD4^+^Treg cell generation/migration into the lymph node [13]. In our findings, hCD8^+^Treg therapy significantly induced mouse self-mCD4^+^Foxp3^+^Tregs and mCD4^+^IFN-*γ* ^+^T cells in the spleen. However, studies on the role of endogenous IFN-*γ* yielded conflicting results. Mice lacking functional IFN-*γ* were found to develop more severe CIA [20], and IFN-*γ* can reduce the number of available osteoclast precursors [21] and inhibit the IL-1*β*-mediated production of MMP1 and MMP3 to limit cartilage degradation [22]. Furthermore, IL-17 differentiation is suppressed by IFN-*γ* [23]. Our results obviously showed that hCD8^+^Treg treatment could significantly reduce the mRNA expression of IL-17A, RANKL, and MMP1 in CIA mouse feet. As reported in a previous study, downregulated IL-17A promoted inflammation by enhancing the production of RANKL to control damage to the synovium [24].

In summary, we have identified and characterized a novel stable subset of polyclonal human CD8^+^Tregs that were induced and expanded by TGF-*β*1 and RAPA. These induced hCD8^+^Tregs expressed CD103 and Foxp3, did not secrete IL-17A, were stable in inflammatory conditions, had potent suppressive ability, and could alleviate collagen-induced arthritis. The induction and amplification of polyclonal human CD8^+^CD103^+^Foxp3^+^ cells might be a therapeutic strategy to help control autoimmune diseases.

## Data Availability

All data are provided in full in Results. We hope readers can access the data from Results to understand the conclusions of the study.

## Supplementary Materials

Supplementary Materials Supplementary Figure 1 (S1): FACS representative data for Figure 2(a). Supplementary Figure 2 (S2): FACS representative data for Figure 2(b).
