# Supplementary material for: Efficient Therapeutic Function and Mechanisms of Human Polyclonal CD8+CD103+Foxp3+ Regulatory T Cells on Collagen-Induced Arthritis in Mice
Source: J Immunol Res. 2019 Feb 19;2019:8575407. doi: 10.1155/2019/8575407 (PMC6399536; doi:10.1155/2019/8575407)

Supplementary Figure1 (S1, FACS representative data for Figure2A)

S1-for Fig 2A

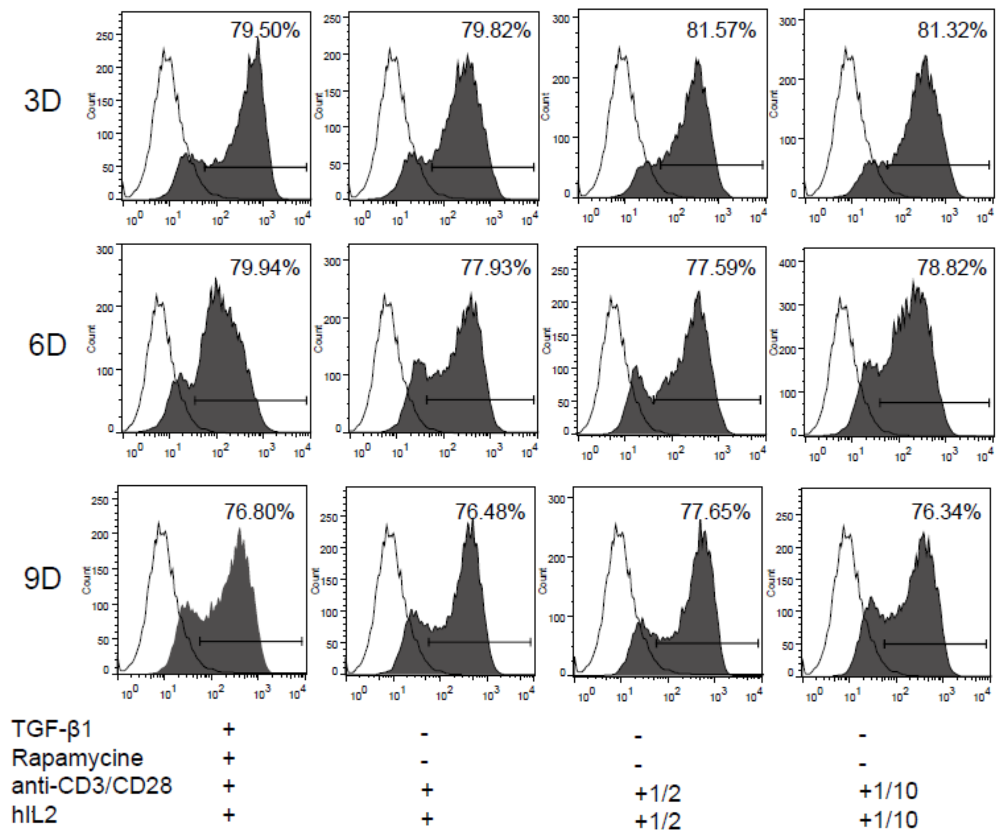

Supplementary Figure2 (S2, FACS representative data for Figure2B)

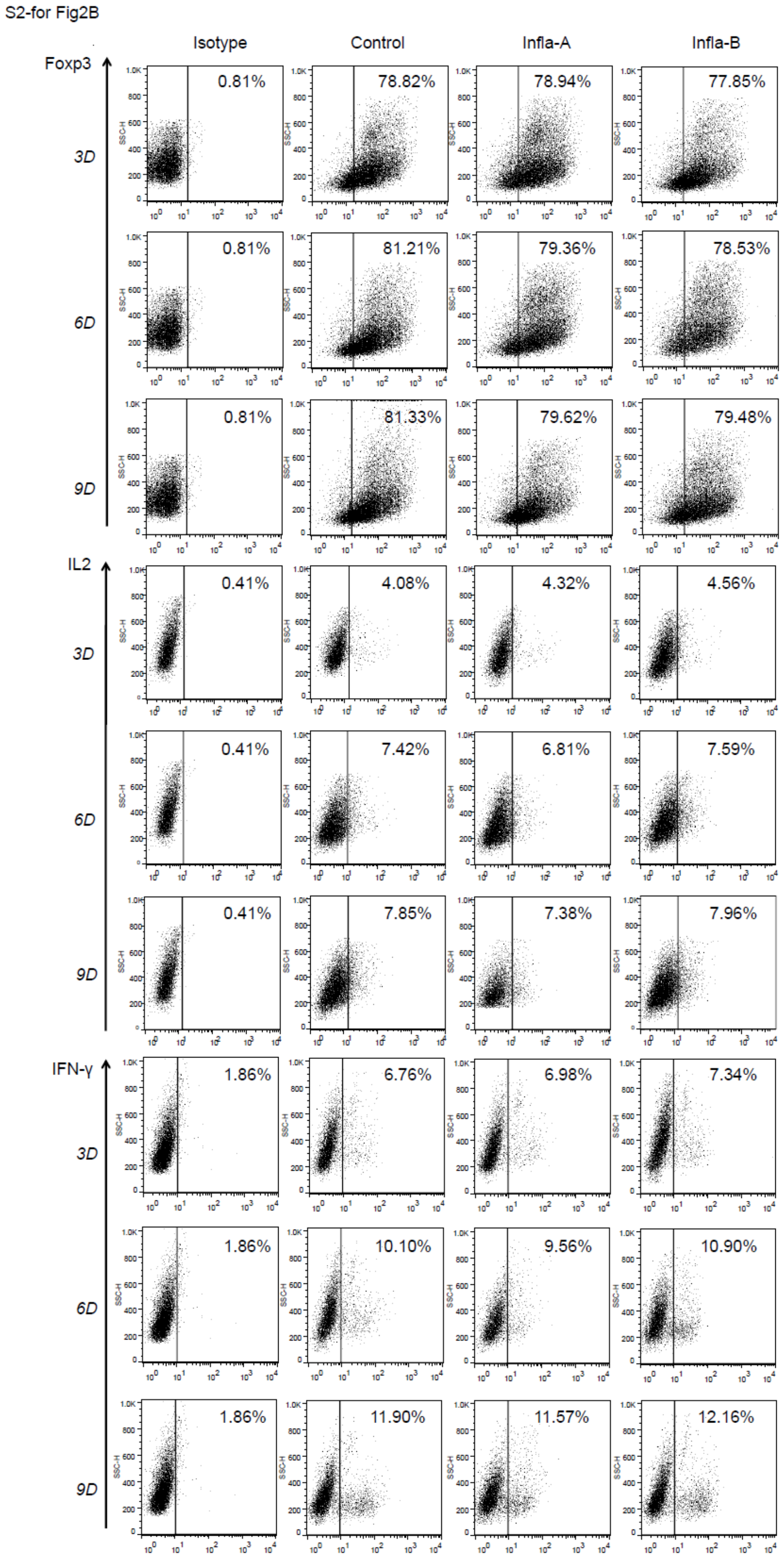

Supplement: Supplementary Materials — Supplementary Figure 1 (S1): FACS representative data for Figure 2(a). Supplementary Figure 2 (S2): FACS representative data for Figure 2(b). [file 8575407.f1.pdf]
